# Artificial intelligence maturity model: a systematic literature review

**DOI:** 10.7717/peerj-cs.661

**Published:** 2021-08-25

**Authors:** Raghad Baker Sadiq, Nurhizam Safie, Abdul Hadi Abd Rahman, Shidrokh Goudarzi

**Affiliations:** 1 Center for Software Technology and Management, Universiti Kebangsaan Malaysia, Bangi, Selangor, Malaysia; 2 College of Business Informatics, University of Information Technology and Communications, Baghdad, Iraq; 3 Center for Artificial Intelligence Technology, Universiti Kebangsaan Malaysia, Bangi, Selangor, Malaysia

**Keywords:** Artificial Intelligence, Maturity model, Systematic literature review, Organization

## Abstract

Organizations in various industries have widely developed the artificial intelligence (AI) maturity model as a systematic approach. This study aims to review state-of-the-art studies related to AI maturity models systematically. It allows a deeper understanding of the methodological issues relevant to maturity models, especially in terms of the objectives, methods employed to develop and validate the models, and the scope and characteristics of maturity model development. Our analysis reveals that most works concentrate on developing maturity models with or without their empirical validation. It shows that the most significant proportion of models were designed for specific domains and purposes. Maturity model development typically uses a bottom-up design approach, and most of the models have a descriptive characteristic. Besides that, maturity grid and continuous representation with five levels are currently trending in maturity model development. Six out of 13 studies (46%) on AI maturity pertain to assess the technology aspect, even in specific domains. It confirms that organizations still require an improvement in their AI capability and in strengthening AI maturity. This review provides an essential contribution to the evolution of organizations using AI to explain the concepts, approaches, and elements of maturity models.

## Introduction

Artificial intelligence (AI) is considered the lifeblood of an organization in different industries. It is a disruptive technology that changes the conventional manner of working in an organization sustainably and radically (Gentsch, 2018). Moreover, besides structured data, AI can deal with data uncertainty (*i.e*. incomplete data or inadequate documentation), making it widely used to solve engineering and industrial problems (Abdullah & Ahmad, 2015; Kiat, Malek & Shamsuddin, 2019). There are various technologies under AI, including machine learning, deep learning, natural language processing models, computer vision, and machine reasoning, which can produce this multimodal knowledge (Dahlan, 2018). Thus, AI could be defined as the ability of a system to correctly interpret external data, produce knowledge from those data to accomplish specific goals and tasks through resilient adaptation (Haenlein & Kaplan, 2019; Kurniawan et al. 2018).

In the Fourth Industrial Revolution (4IR) era, with exponentially increasing information assets in organizations (Bakar, Razali & Jambari, 2020; Hassan, Yusof & Ahmad, 2019) and the need for measurements and forecasting future values (Khaki et al., 2016), many organizations are investing in adopting AI to increase their capability. The importance of intelligence originates from its ability to add a competitive advantage to an organization over its rivals, create added value through enhance performance, productivity, effectiveness with low cost, increase the work of executives and staff, speed up the process, and cater better to customers (Gentsch, 2018; Lichtenthaler, 2020; Seger, Miailhe & Mueller, 2019; Zhang, Song & He, 2020). Despite these benefits of AI, some organizations are still far from applying AI to their businesses or their value chain (Lichtenthaler, 2020). Some have adopted AI technologies in their business but are still at an early stage, with limited benefits achieved (Mikalef, Fjørtoft & Torvatn, 2019; Nortje & Grobbelaar, 2020). For example, Lamberti et al. (2019) surveyed to reveal whether AI in the pharmaceutical industry is mature or not. The results revealed that the AI maturity overall was assessed at the nascent level by 75–100% of the highest rating respondents. Besides that, two out of five considered AI functions are immature due to the substantial unrealized potential focused on an integrated intelligence architecture for further improving their competitive positions (Lichtenthaler, 2020). In other words, the implementation of AI should be organization-wide; otherwise, it will achieve a fraction of the realizable value (Seger, Miailhe & Mueller, 2019).

Organizations face various challenges, such as implementation costs, insufficient volume of relevant data, and misalignment of strategic goals, whereby the prominent challenges are typically human-related issues (Burgess , 2018). Besides that, organizational implementation, specialization and expertise, AI safety (trust, privacy, and ethical concerns), no governance to direct the process, inertia (reluctance to change work practices), infrastructure, and lack of top management support are additional business challenges and concerns in the transformation process. The latter also revealed that the most significant challenges in implementing and adopting AI are safety and data, followed by specialization, expertise, and inertia (Kılınç & Ünal, 2019; Mikalef, Fjørtoft & Torvatn, 2019). All above indicate that organizations face a great challenge during the implementation and integration of AI into their businesses, and part of this includes the maturity and readiness of the business to apply AI (Saari, Kuusisto & Pirttikangas, 2019). Even organizations that are considered “AI leaders” and constantly improve AI capability and create more value still do not leverage their organization’s full AI strategy space or not in large parts of their organizations (Lichtenthaler, 2020). Thus, the business that invests in AI implementation ensures long-period survival and improved performance, while the others will be eliminated in the near future (Lamberti et al., 2019).

To help organizations find a path to enhance their performance and meet their objectives by evaluating their capability, the notion of the “maturity model” was invented. Maturity models are action-oriented standards that consist of discrete maturity levels for a class of processes or organizations. It represents the stages of increased quantitative or qualitative capability changes of these processes or organizations to evaluate their progressions concerning defined focus areas (Becker, Knackstedt & Pöppelbuß, 2009; Dzazali & Zolait, 2012; Fraser, Moultrie & Gregory, 2002; Kohlegger, Maier & Thalmann, 2009). This helps users understand the requirements to change and take the appropriate steps to accompany the change process (Bahri & Fauzi, 2018; Felch, Asdecker & Sucky, 2019). Mohammad et al. (2009) emphasizes the importance of assessing their current business excellence maturity level as a critical factor in its model to choose the right initiatives that lead to increased efficiency, productivity, and sustainability to achieve excellence. The Capability Maturity Model (CMM) was released in the early 1990s to evaluate Software Process maturity in organizations (Abd Hamid & Mansor, 2016; Paulk, 2009). It serves as a reference model for software process improvement (SPI) efforts in hundreds of software companies across the world (Herbsleb & Goldenson, 1996). Since then, plentiful maturity models were developed in various domains to assess competency (De Bruin et al., 2005). Maturity model research has been introduced in more than 20 fields, but software development and software engineering disciplines are still heavily dominated (Wendler, 2012). Table 1 summarizes some of the previous reviews related to different maturity models.

**Table 1:** Summary of published reviews on maturity models.

Author	Approach	Scope	Characteristics
Ochoa-Urrego & Peña-Reyes (2021)	Three stages planning, conducting, and reporting, and dissemination) was proposed based on Tranfield, Denyer & Smart (2003) related to different layers of business architecture.	-16 articles -Scopus, EBSCO, ProQuest, and Web of Science -Six models and a model presented by the Ministry of ITC in Colombia.	The framework used for model design is the multidimensional orientation. However, the biggest interest is oriented towards the strategy and business layers which cannot guarantee any transformation process.
Lasrado, Vatrapu & Andersen (2015)	Five main steps starting from identifying raw keywords search until to selecting the relevant papers in IS domain.	- 34 articles within 1999 to 2014 - IS conference proceedings, books in ACM, AIS electronic library, IEEE explore, Springer link, and Business source databases.	A few maturity models that included theory, causal approaches, or hypothesis testing, the bulk of maturity models are conceptual in nature. The analysis demonstrates empirical validations of the models are scarce.
Santos-Neto & Costa (2019)	Ten main steps from PRISMA (Moher et al., 2015) are used in classification framework to analyze and structure the selected publications (Tarhan, Turetken & Reijers, 2016; Webster & Watson, 2002; Wendler, 2012) and investigate the maturity models in different enterprise segments.	- 409 articles until 2017 - ScienceDirect, SpringerLink, Scopus, Web of Science and lecture -Related to development, validation, or application of maturity models.	The studies are more descriptive approaches than prescriptive ones, focusing on the bottom-up approach. The level of maturity in most development and application papers was defined according to a scoring system.
Bertolini et al. (2019)	A combination of qualitative and quantitative was utilized to comprehend maturity model trends and topics in general and the specific subjects and characteristics of maturity models.	- 65 articles - 9 articles are related to maturity models obtained from Scopus.	The majority of the literature falls under the Manufacturing and Information Systems subject categories. Therefore current maturity levels are focusing on socio-cognitive factors than on objectively measuring technology and knowledge stack.
Tocto-Cano et al. (2020)	Four main steps from (Keele, 2007) are used from process of identifying research questions until the systematization to evaluate maturity in university education domains.	- 23 articles between 2007 and 2020 - Scopus, Web of Science, ScienceDirect, IEEE, ERIC, EBSCO Discovery, and Wiley.	Nine categories were organized the selected articles based on their purposes. However, maturity models used in universities move towards agility, automation through ontologies and the semantic web; lack of guideline of best practices.

**Note:**

A summary of most related review papers on maturity model according to their approaches, scopes and characteristics.

One of these areas for which maturity models have been developed is AI. AI domain maturity models are valuable tools used to define the degree of ‘readiness’ to take advantage of AI (Saari, Kuusisto & Pirttikangas, 2019) by improving organizational performance to evaluate and enhance AI-created capabilities (Alsheibani, Cheung & Messom, 2019). An AI maturity model (AIMM) is used to assess intra-organizational AI capabilities (Coates & Martin, 2019), to guide in the AI journey (Burgess , 2018), and as a comparison of AI capabilities within an organization or with progressive organizations (Alsheibani, Cheung & Messom, 2019; Saari, Kuusisto & Pirttikangas, 2019). Thus, the organization should systematically identify its needs and abilities to create a prioritized portfolio of initiatives for AI (Saari, Kuusisto & Pirttikangas, 2019). While doing so, managers should evaluate the impact, feasibility, objectives alignment (ambitious), and availability of technology (Burgess , 2018; Saari, Kuusisto & Pirttikangas, 2019). To assess AI maturity, all the underlying AI dimensions (*e.g*., data, AI culture, etc.) of specific or all areas (*e.g*., automation, marketing, etc.) in an organization should be evaluated to obtain a result in terms of the total level of AI capability (Mikalef, Fjørtoft & Torvatn, 2019). In addition, the maturity models should be developed based on the theoretical foundation because the conceptualization of maturity models may be affected by various theoretical perspectives (Lasrado, Vatrapu & Andersen, 2015). Various AIMM were developed with different dimensions to evaluate and increase firm-wide AI capability or in specific areas of the firm. The purpose of this study is to conduct a systematic literature review (SLR) to provide an overview of the state-of-the-art AIMM, identify gaps in the literature, and identify opportunities for future work.

## Survey methodology

**Figure 1:**
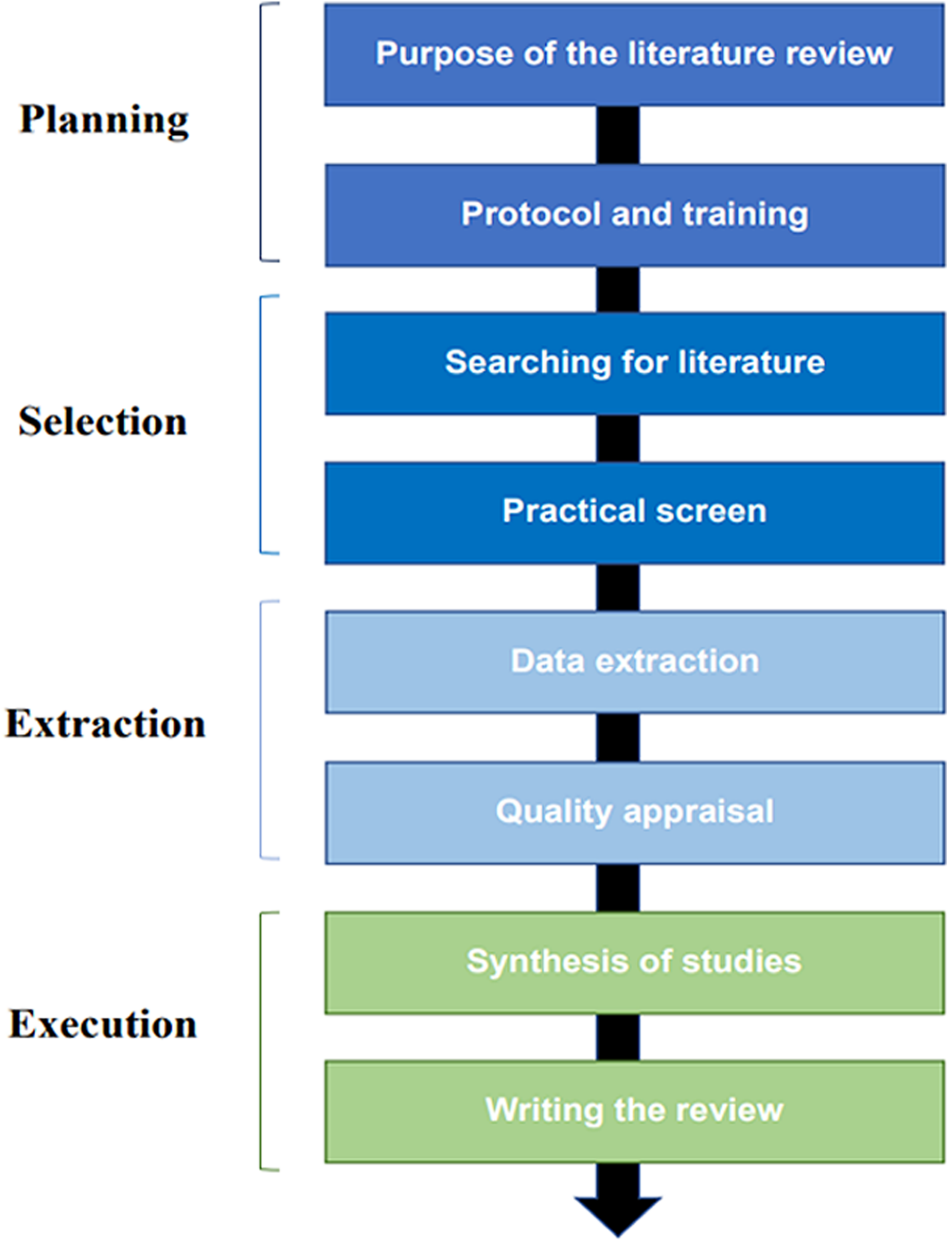
The methodology of the systematic literature review. Four phases of the systematic literature review containing eight main processes.

This work implemented a SLR to retrieve all the relevant research that targets AI and maturity models. This is attributable to its rigorous approach to provide answers to specific questions or to test particular hypotheses (Greenhalgh & Peacock, 2005; Okoli, 2015). Therefore, extensive literature reviews have been performed to provide a thorough understanding and critical evaluation of the maturity models relevant to AI, besides finding new paths for research through identifying gaps. An eight-step guideline by Okoli (2015) is performed to help researchers enhance systematic reviews in information science (Fig. 1). It covers (i) Purpose of the literature review; (ii) protocol and training; (iii) searching for literature; (iv) practical screen; (v) data extraction; (vi) quality appraisal; (vii) synthesis of studies and (viii) writing the review.

These eight steps are applied in the subsequent sections for a study of the AIMM. “Survey Methodology” describes steps (i) until (v), followed by steps (vi) and (vii) in “Synthesis Studies”. Step (viii) is addressed in “Discussion”. Finally, “Conclusions” summarized the outcomes of this review.

### Purpose of the literature review

The maturity model is used as a scale for assessing the position on the path of evolution (Becker, Knackstedt & Pöppelbuß, 2009). The position is identified by assessing the competency, capability, and level of sophistication of an organization in a specific domain, based on a set of criteria that is more or less comprehensive (De Bruin et al., 2005). Each level or stage describes criteria and characteristics that need to be satisfied to achieve a specific level of maturity (Bahri & Fauzi, 2018; Becker, Knackstedt & Pöppelbuß, 2009). In addition, the description of the criteria and characteristics in the next level could be used as best-practices guidelines on how to reach a higher level (Fraser, Moultrie & Gregory, 2002).

Alsheibani, Cheung & Messom (2019) mentioned that the academic sources in this topic are scarce, and most of them are produced by analyst organizations, besides the theoretical background is missing. The same conclusion was reached by Ellefsen et al. (2019), who indicated that the AIMMs are established in a limited number of articles from Scopus or Web of Science databases. Various studies related to AIMM were conducted with different objectives. An evaluation is usually performed using a maturity model when organizations need to assess how far they are becoming an “AI-first” organization (Gemmink, 2019). A number of AIMM were developed to specify the degree of preparedness to benefit from AI in terms of improving organizational operations and processes, introducing new AI technologies, linking to the state of the company and its mindset, and estimating the performance of the organization enabled by AI technology (Saari, Kuusisto & Pirttikangas, 2019). Besides, these models must be tested for rigor and thoroughness to ensure their ability to be a well-rounded tool to reflect the current state of AI capability. This acted as a motivation for the following research questions:

**RQ1:** What are the main objectives in the literature on maturity models in the AI domain?

**RQ2:** What are the research methods used in the literature on maturity models in the AI domain?

**RQ3:** What is the scope of maturity models in the AI domain?

**RQ4:** Which design approach, typology, architecture, purpose of use, and components are used to design maturity models in the AI domain?

### Protocol and training

A strategic search strategy was used for analyzing and extracting relevant data. The protocol defined the criteria for inclusion, exclusion, keywords, the source databases, and quality assessment to ensure accuracy and consistency across the reviewers and papers. This step is necessary, regardless of the number of reviewers working on a study.

### Searching for literature

During the search process, eight academic digital sources were selected to extract relevant studies from the literature. Expert advice and prior systematic analysis were considered in this study to determine the database sources to be used. These databases cover the latest reliable literature topics and studies across all domains. The seven bibliographic databases considered are Scopus; Web of Science (WoS); Dimensions.ai; SpringerLink; ACM Digital Library; IEEE Xplore; and Pro-Quest. Another source, the academic search engine, Google Scholar has also been used since it is still the most comprehensive source (Martín-Martín et al., 2021). For the search string, the keywords used were “Artificial intelligence”, “AI” and “maturity” to search for relevant papers. During the process, Boolean operators were utilized to gather literature that is as pertinent as possible. To narrow down and retrieve relevant results, “AND” and\or “OR” operators were used for databases and the search engine. In SpringerLink and Pro-Quest, the “NEAR” operator was used, which is equivalent to “AND”.

The same approach is used to find articles where the keywords joined by the operator are within a certain number of words of each other. Since each search engine has its own way to work (*e.g*., search field and operators), each search string was changed to find the preferred results without reducing the validity of the research by missing relevant references. The changed search string was represented that previously defined in the SLR protocol. For this reason, in ACM Digital Library and IEEE Xplore, the searching process was conducted in abstracts (the most suitable choice) due to the limits of these search engines’ choosing and the topic of research always be mentioned in the abstract. This study used the default number of words describing the allowed separation (10 in SpringerLink, 4 in Pro-Quest). Using this helpful operator in the two databases is due to the popularity of AI as a topic, and there are numerous studies related to it. In addition, “maturity” is a widespread word, and many studies use it as it is generalizable across many domains. Besides that, the two latter databases contain books, theses, and dissertations that consist of mostly a hundred pages, which is costly in terms of time and effort. Therefore, by using “NEAR”, only the relevant papers would be retrieved from the databases. On the other hand, besides “NEAR” and due to its functionality, the “OR” operator is included to ensure that we did not miss any important research that our mapping is seeking to address. In Google Scholar, the number of results was huge, and a high proportion of them was not relevant. Perhaps because it indexes the entire text of every article or recall papers that discussed only the main concepts in the full text (Martín-Martín et al., 2021). Thus, to minimize the results by retrieving only the relevant papers, the search string included “OR” operator. On the contrary, in the rest of the databases, the “OR” operator retrieved irrelevant sheets, which it became necessary to delete because the AI is short for other words like agile. All the academic literature was selected for the publication types, including journal articles, conference papers, book chapters, dissertations, and theses. Therefore, to extract only the relevant materials, the search string was modified with the exact phrase. To define the state-of-the-art papers, only published papers after 2015 until September 2020 and written in English were selected. The databases and the search engine with their queries are listed in Table 2.

**Table 2:** The databases and string searches used in this study.

Sources	Search String
Scopus	(“Artificial Intelligence” AND “Maturity”)
WoS	(“Artificial Intelligence” AND “Maturity”)
Dimensions.ai	(“Artificial Intelligence” AND “Maturity”)
SpringerLink	(“Artificial Intelligence” OR “AI”) NEAR (“Maturity” )
Google Scholar	(“Artificial Intelligence Maturity” OR “AI Maturity” OR “Artificial Intelligence Process Maturity” OR “AI Process Maturity”)
ACM Digital Library	(Abstract: “Artificial Intelligence”) AND [Abstract: “Maturity”]
Pro-Quest	(“Artificial Intelligence” OR “AI”) NEAR (“Maturity”)
IEEE Xplore	(“Abstract”: “Artificial Intelligence”) AND “Abstract”: “Maturity”)

**Note:**

Each of the sources containing different search strings to enhance the search outcomes.

#### The screening I: title

The metadata with the date of extraction was collected with their significance labeled. Initially, all the results of the databases and Google Scholar were reviewed for duplication resulting 493 papers selected from 686 for further investigation. The number of studies initially retrieved and selected in the databases and Google Scholar are shown in Table 3. In this review, the title of each paper was scanned and identified for possible relevance to this review. Any paper that does not refer to AI-related maturity was eliminated from further investigation. After the initial search and screening of the 493 specific studies, 84 important papers were found.

**Table 3:** Articles screening process from each databases.

Sources	Initially retrieved	Initially selected	Records screened I	Records screened II	Final selection
Scopus	241	241	26	10	5
WoS	57	17	4	2	0
Dimensions.ai	125	36	5	2	2
SpringerLink	119	108	24	4	3
Google Scholar	72	54	21	10	5
ACM Digital Library	8	0	0	0	0
Pro-Quest	43	33	3	1	0
IEEE Xplore	21	4	1	0	0
**Total**	**686**	**493**	**84**	**29**	**15**

**Note:**

The number of articles that were included in each step of the screening process from different databases.

#### The screening II: abstract and keywords

In the second screening process, the abstract and keywords of the publications were scanned for a deeper review and a better understanding of the papers that focus on maturity models-related AI. The inclusion criteria in this review are the studies that:

- refer to a maturity model that focuses on the AI domain (introduce, propose, develop, and validate).
- apply an AI maturity model to assess AI in an organization.
- publish as journals articles, conference papers, theses, dissertations, book chapters, and reports.
- are written in English and published after 2015.

On the other hand, the exclusion criteria used in this review are as the studies that:

- referring to a maturity model that focuses on a particular aspect of AI or a related field like machine learning, deep learning, or data maturity models.
- introduce or develop a method for assessing the AI capabilities’ level of maturity in an organization, but do not refer to a generic model that meets the maturity model definition.
- mention AI maturity model or the keywords without enough information.
- refer to maturity models in domains other than AI.
- include experience papers, forums, comments, tutorials, opinions, or discussions.

To avoid losing potential contributions for the research topic, we decided to preserve all papers (not just peer-reviewed papers). A total of 29 studies met the inclusion criteria, as shown in Table 3. Another round of screening was applied to these articles examining their full text for quality appraisal of the elicited articles from the previous stage.

#### Quality appraisal

The papers included in the second screening were fully read to determine their emphasis on AI relevant to maturity models and their importance to the research questions. These papers were analyzed accurately with a concentration on their aims, description, and methodology. A summary of information from the sample papers are filtered in the final screening stage on an excel sheet. Each part of this sheet contains details that would answer a specific research question. From this process, 14 articles were excluded, and a final sample of 15 primary papers conformed to all criteria were considered convenient for inclusion in this review.

#### Data extraction

For AIMMs, prominent information was extracted, such as assessment models about procedures and methods used for these models, scope, and metrics. The final set of papers in this review show different attention to this information; some papers devote considerable effort, while others apply far less care to explain the details. A classification scheme was achieved to analyze and structure the final set of papers, as shown in Table 4. A set of classes and subclasses were identified for the classification scheme, based on contributions from the literature (Becker, Knackstedt & Pöppelbuß, 2009; De Bruin et al., 2005; Fraser, Moultrie & Gregory, 2002; Frick, Küttner & Schubert, 2013; Mettler, 2011; Wacker, 1998) and research questions. These classes aim to answer the defined research questions. The “N/A” label was used when there was insufficient information about a class in the study.

**Table 4:** Classification scheme of this review.

No	Class	Subclasses	Description
1.	Search objectives	Development	Propose, adapt, or create a new maturity model in the AI domain
		Validation	Validate proposed or existing conceptual models
		Application	Deploy AI maturity models and assess the AI maturity of organizations.
2.	Scope	AI domain	“General” or “Domain Specific” models for examining the maturity of AI
		AI analysis level	The AI level of analysis can be a company and/or department, project, system, and process
3.	Research method	Analytical	Either conceptual, mathematical, or statistical methodologies
		Empirical	An experimental design like case studies, content analysis, or mixed methods
4.	Design approach	Top-down	During the design process, the maturity levels were first established, and then the items of assessment.
		Bottom-up	The assessment item was first identified during the design phase and then the maturity levels.
5.	Architecture	Stage	A cumulative set of activities included in each level have to be successfully accomplished before moving to the next level.
		Continuous	A collection of activities which can be separately approached.
6.	Purpose of use	Descriptive	It will provide a deeper understanding of the *status quo* of AI and establishes the level of quality of AI domain at a given time.
		Prescriptive	It shows a roadmap for improvement, or it identifies the strategy and practices required to progress to the next maturity level.
		Comparative	It has mechanisms of comparison and is benchmarked with other domains.
7.	Typology	Maturity grids	A matrix contains a number of maturity levels that attend to different stages of AI capabilities.
		Structured models	A structure that is formal and complex, like the CMM
		Likert-like questionnaires	A series of questions in which the respondent identifies the level of AI maturity of the organization on a scale from 1 to n.
		Hybrid	Combination use of matrix grid and Likert-like questionnaires.
8.	Components	Description of level	If there is an explanation of the characteristics of each level, the value ‘Yes’ is used, or else ‘No’ is used.
		Elements	A set of practices /activities/capabilities that are used to analyse or evaluate the maturity level and lead to achieving a set of goals considered to reach higher levels of maturity.
		Descriptors	The distinguish name for each maturity level
		Names of maturity levels	Labels of levels
		No. of levels	The number of maturity levels as the degree of maturity

**Note:**

The classification scheme of maturity models are describes according to their subclasses.

## Synthesis studies

In this section, all 15 papers identified from the literature were analyzed to obtain a deeper understanding of the topic and answer the research questions.

### Sample description

**Figure 2:**
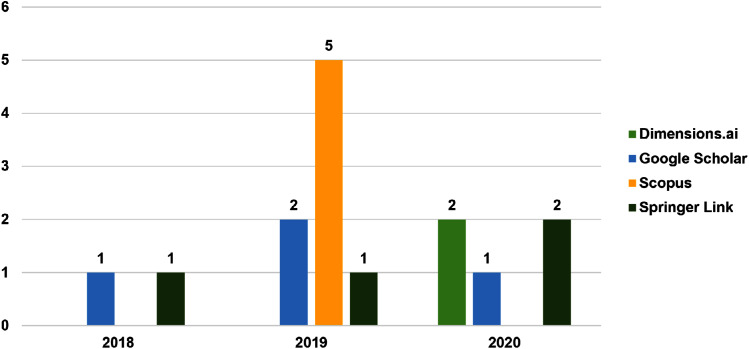
Distribution of articles per database and year. Each data indicates the total of publication published according to databases from 2018–2020.

**Figure 3:**
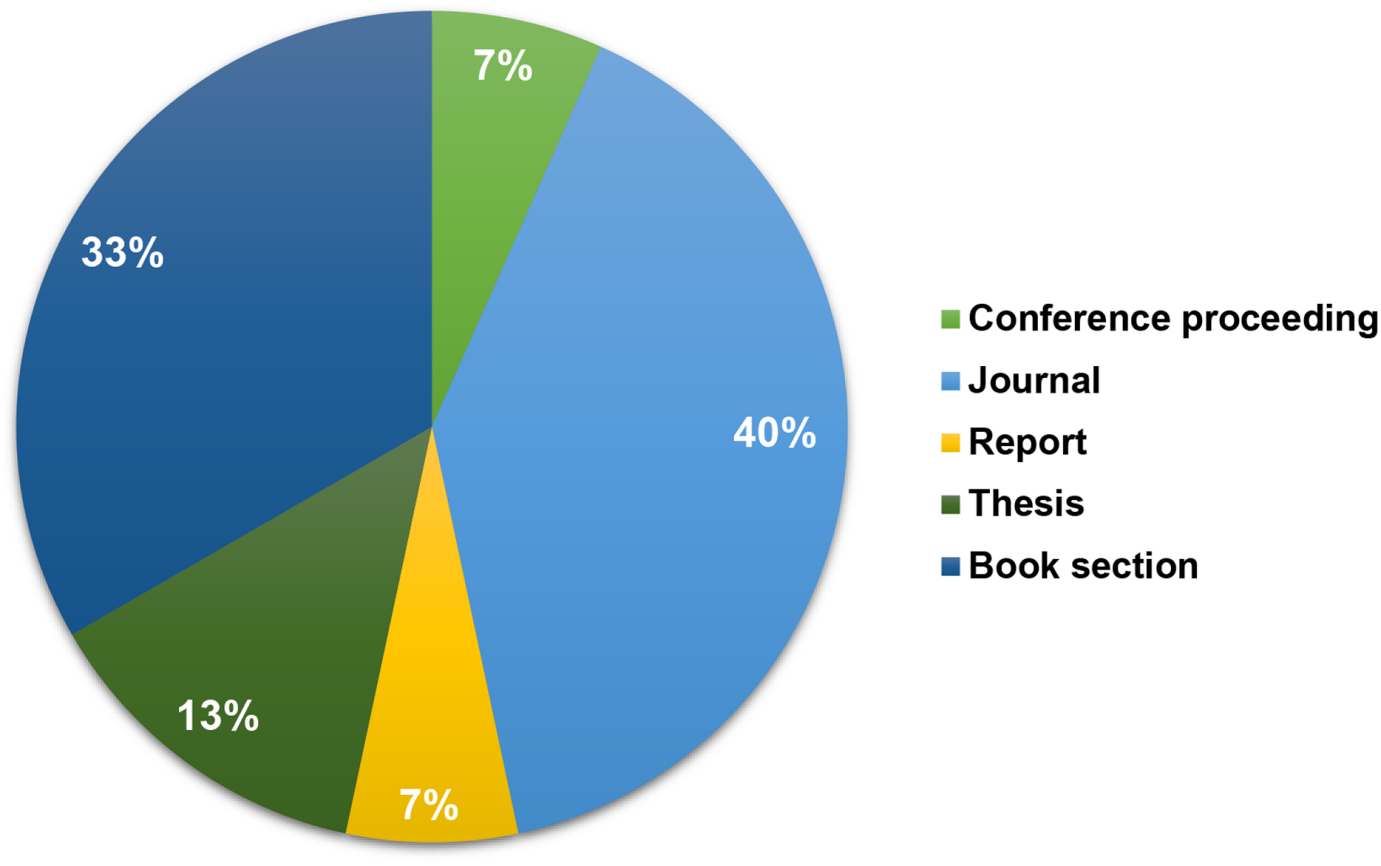
Distribution of articles by publication type. The percentage values (%) indicate the distribution of all related articles per publication category.

Three aspects of the main demographic statistical results from the papers are discussed, namely, sources, publication types, and the year of publications, as shown in Figs. 2 and 3. The distribution over databases and Google Scholar discovers that Scopus held most of the publications on this topic. The literature search performed in this review started in 2016, showed that no paper on this topic was published in 2016 and 2017. The findings in this review is summarized in Figs. 2 and 3.

### Research objectives and methods

The studies are classified into two categories, namely, analytical and empirical papers. Our analysis reveals that half of the studies approximately performed validation for the maturity models. There are seven conceptual studies wherein no testing or application of the existing models was performed. In terms of model validation, seven out of the 15 models were validated using various empirical research approaches. The validation phase is important to ensure the proposed model’s comprehensiveness, consistency, and problem adequacy (Becker, Knackstedt & Pöppelbuß, 2009). Concerning the validation, only two article used a quantitative survey method, with more than 80 respondents based in Finland. The results show that many organizations have only just started exploiting AI in their organizations. Only three papers out of seven implemented qualitative methods. Jaaksi, Koskinen & Jalava (2018) conducted interviews with 11 experts to validate the model. The highest proportion of validation approaches are mixed methods, such as in Coates & Martin (2019), who used a mixed research method by performing a quantitative survey followed by case studies which analyzed the results quantitatively and qualitatively. Ellefsen et al. (2019) combined survey instruments with multi-case studies that were conducted through interviews. Gentsch (2018) analyzed the content available on the internet of three organizations, with an extremely high level of automation. Regarding the development phase, the majority of the papers used qualitative methods (*e.g*., action research or case studies), but the dominant method is content analysis. The “Application” column is empty, which means no research has applied any existing AIMM.

### Main characteristics and components of AIMMs

This section discusses the main characteristics and components of AIMM. Wendler (2012) indicated that not all the selected research had a clear definition of the term “maturity model” and this is also seen in our chosen studies. Only two studies out of 15 reviews contain different definitions of the term. The variation of the definitions proves that there is no consensus on a unified definition of the term ‘maturity model’. The definitions of the maturity model in our findings studies are (i) an essential tool for describing existing production capabilities (Lee, 2020) and (ii) a measurement tool to evaluate the capabilities of an organization (De Bruin et al., 2005). Our findings were analyzed according to the classification in Table 4.

According to the components, the elements in the maturity models under study have different designations, including “dimensions” (Lamberti et al., 2019), “constructs” (Mikalef, Fjørtoft & Torvatn, 2019), “elements” (Gemmink, 2019), “indicators” (Ellefsen et al., 2019), and “process areas” (Kumar, 2017). These findings reveal that these elements have different naming, and no standard terminology is available. This is in line with Poppelbub & Roglinger (2011), who reached the same conclusion. These elements are considered the heart of any maturity model, because their different capabilities represent the maturity levels. However, not all the selected models illustrated them clearly. Four or five levels are indicated in two models, without information about the dimensions used for evaluation. The authors define the principal components associated with each maturity model. The 15 research studies under review are explained briefly below in alphabetical order of the title. The information in all tables has the same order to simplify reading and comparison.

*Study 1:*Gentsch (2018) aimed to develop an AIMM for the marketing domain based on the AI business framework. Four levels of maturity, from the non-algorithmic enterprise to the automated enterprise, and finally super intelligence enterprise, the model showed the various stages of development regarding data, algorithms, and AI. Although the author included the “superintelligence enterprise” level as the highest maturity level, he indicated that it is difficult to reach this level at present. The model contained five dimensions: strategy, data, analytics, people/Orga, and decisions. However, there is no information about how these dimensions are evaluated to measure maturity.

*Study 2:*Kreutzer & Sirrenberg (2020) described an AI maturity map to determine the extent of existing AI and provide a path to start the AI journey in an organization. The analysis included two parts: AI basics and AI fields of application. Each part was evaluated separately by percentage value (a range from 0–20% to 80–100%). The four dimensions of AI basics should be evaluated for all organizations. Although the areas of application identified in this study were customer service, marketing/sales, service delivery, and production, it could decrease the current areas or include others like maintenance, human resources, and related fields based on the organization’s focus. However, dimensions are not described at each level and there is no information about how these dimensions are evaluated to measure maturity.

*Study 3:*Saari, Kuusisto & Pirttikangas (2019) developed a free web tool to assess AI capability in a company context. The tool was developed by AI Accelerator (FAIA) in Finland, where the AI maturity index was first published in Finnish. This tool can be employed to evaluate the maturity in an organization to improve the understanding of the AI maturity level in different departments of the organization and possible areas of development, which is a valuable gauge before any development projects are launched. The model has six dimensions: strategy and management, products and services, competencies and cooperation, processes, data, and technology. The assessment method is carried out through questions, with five response alternatives to which each dimension has two questions. However, dimensions are not described at each level.

*Study 4:*Coates & Martin (2019) developed a new free self-assessment instrument reflecting the ethical values by actionable methods that would assess organization maturity to control AI bias efficiently. The instrument was designed to evaluate the project through two stages: Design and Development (during the project development) and Post-Development (after finishing the project’s development). During the first stage, the evaluation can be performed multiple times, with the expectation that companies can increase their maturity after each sprint. The constructs used in the design and development phase are business, people, user, data, algorithm, and compliance, and for the post-development phase are business, data, test, client feedback, and transparency. The maturity gain is a score as the sum of the relevant question’ answers, divided by the maximum possible score, to give a proportion of performance independently. However, there is no evaluation of maturity levels after the scores to understand the level reached.

*Study 5:*Abele & D’Onofrio (2020) suggested an AI maturity to assess intelligence systems’ intelligence capabilities. It provides a way to assess the level of technology growth in relation to one another or measure performance in a specific context. The model involves a two-dimensional classification where the x-axis represents the ability to deal with uncertainty, while the y-axis reflects the capability to solve problems adaptively. The adaptive method solves problems and shows the ability of a system to be applied in totally different contexts. The model shows that AI systems could be classified into five independent discrete categories. But there is no explanation on how to evaluate these systems.

*Study 6:*Tu, Lin & Lee (2019) proposed a metadata-driven automation maturity model for a clinical research company. The model intends to help these companies evaluate their processes’ intelligent automation depending on their integration, efficiency, and intelligence. Three indicators were used to assess the maturity, which are: standard adoption (SA), code reusability (CR), and process repeatability (PR). All indicators’ capabilities were described using nine maturity levels.

*Study 7:*Bangalore Seetharam (2020) presented a digital AI maturity ladder for retail organizations in different industries. The ladder was developed to enable them to know the current state of AI and offer a guideline to increase AI adoption against time, depending on the organization’s ambition. The ladder consists of ten steps with four drops (stages), whereby each step defines the subsequent progression in becoming an AI-driven organization and gaining better value. Drop 2 is considered the start point to embark on AI. There are generic descriptions for drops with descriptors, but the capabilities used to evaluate the drops were not clarified. The author mentions that the evaluation was conducted through interviews.

*Study 8:*Lichtenthaler (2020) proposed a conceptual framework with three elements used to construct the five levels of maturity to manage AI in a company and helps to recognize the managerial challenges and significant resource limitations of organizations. The three elements are various types of AI, various types of human intelligence, and the meta-intelligence to guarantee that the final architecture is more than the aggregate of the different types of intelligence. This framework was built on the grasp of intelligence architecture based on the concept of Integrated Intelligence and the intelligence-based view of company performance.

*Study 9:*Jaaksi, Koskinen & Jalava (2018) introduced a model that assesses the current maturity level of AI adoption in organizations to address the requirements of AI transformation. The result indicated that the most important five dimensions were correlated with the AI maturity of the company. Feasible quality and quantity of the data to be used to implement the designed solution, workers who have the expertise and skills to create the designed solutions internally or successfully. The state of the organization with the current implemented solutions and the organization’s mentality to adopt AI solutions. All capabilities of dimensions were indicated in four levels with detailed descriptions.

*Study 10:*Lee (2020) proposed a maturity capability assessment method of crucial technologies in Industrial AI. The objective is to create a thorough assessment of an enterprise’s level of Industrial AI development to provide a foundation for its decision-maker and motivate to improve and address its deficiencies. By matching the four elements with the intelligent transformation stages, the level of industrial AI maturity would be determined. The assessment is based on four technologies, namely, Data (DT), Analytics (AT), Platform (PT), and Operation (OT) technologies. He explained each technology’s capabilities separately in several maturity stages (all have four stages, except Analytics, which has five stages). However, the author did not explain the procedure to evaluate these capabilities.

*Study 11:*Yablonsky (2019) used a previous AI automation framework and AI/Big Data (BD)/Advanced Analytics (AA) value framework to produce a multi-dimensional data-driven AI enterprise innovation maturity framework to assist with strategic innovation planning and AI-based automation investment decisions. The framework concentrates on data-driven human-machine relationships and implementing AI at five levels of data-driven automation maturity scope, from activities and tasks to AI processes and platforms. The evaluation method to determine the level does not exist.

*Study 12:*Burgess (2018) suggested an AI maturity matrix and AI heat map to draw the path for an organization to initiation the AI journey, and offers an opportunity to identify the roles and opportunities of AI palpably and automation across the business which are acceptable and technically and economically feasible. Four criteria in the AI heat map (Strategic objectives, Existing challenges, Benefits, and AI capabilities) were used according to their processes in areas (customer service, organization, operations, and finance). All six maturity levels, from level zero to level five were defined and each level comprises related practices for a predefined set of process areas that enhance the performance of an organization. The evaluation process could be determined through interviews and evidence analysis.

*Study 13:*Ellefsen et al. (2019) presented an AIMM framework to measure the AI readiness of logistics companies. The authors developed a survey instrument (questionnaire) to understand and evaluate the current situation of existing solutions of Logistics 4.0 and the implementation of AI in logistics companies. The four maturity levels of AI maturity for communication service providers (CSP), produced by Ovum, were used for AI levels evaluation. The results revealed that all the selected companies were still at a novice level.

*Study 14:*Seger, Miailhe & Mueller (2019) proposed an AI maturity framework for Intergovernmental Organizations (IGO). The framework focuses on executives use, contains five levels of maturity. However, no information about the dimensions or how to apply this framework for evaluation was provided.

*Study 15:*Alsheibani, Cheung & Messom (2019) introduced a conceptual model to evaluate the AIMM to helps an organization to have a vision to effectively develop and implement AI. the dimensions of the model are: AI functions, data structure, people, and organization, to measure maturity. All capabilities were indicated using five levels with a particular descriptor.

This review proves that maturity models in the AI domain were developed with different components. Table 5 reveals that the AIMM has different designs regarding the number of levels, descriptors, and elements. Various descriptors were identified from the 13 models under study, which differ from one model to another. The elements column discovers different designations and that there is no standard terminology. But the majority used “dimensions”, which is mentioned in seven studies. Only three studies are without element identification.

**Table 5:** The components of AI maturity models.

Author	Components				
	No. of levels	Descriptor	Levels description	Names of maturity levels	Elements
Gentsch (2018)	4	Yes	Detailed description	non-algorithmic, Semi-automated, Automated, Superintelligence	5 dimensions (Strategy, Data, Analytics, People/Orga, and Decisions)
Kreutzer & Sirrenberg (2020)	5	Yes	Very brief description	0–20%, 20–40%, 40–60%, 60–80%, 80–100%	2 parts (AI basics and AI fields of applications) subdivided in 8 dimensions (AI targets/strategy, AI budget, AI employees, AI systems), and (Customer service, Marketing/Sales, Service delivery, and Production).
Saari, Kuusisto & Pirttikangas (2019)	5	Yes	No	Does not exist / Not relevant, Preliminary, Defined, Managed, Excellent	6 dimensions (Strategy and management, Products and services, Competences and cooperation, Processes, Data, and Technology)
Coates & Martin (2019)	N/A	No	No	N/A	11 constructs assessed though two stages: Design and development (Business, People, User, Data, Algorithm and Compliance, transparency and accountability), Post-development (Data, Testing, Client feedback and Transparency and accountability)
Abele & D’Onofrio (2020)	5	Yes	Detailed description	Smart information systems, Reactive machines, Artificial Narrow Intelligence, Artificial General Intelligence, Self-aware AI	2 dimensions (Uncertainty and Problem-solving capability)
Tu, Lin & Lee (2019)	9	Yes	Detailed description	ML1,ML2, ML3, ML4, ML5, ML6, ML7, ML8, ML9	3 indicators (Standard adoption, Code reusability, and Process repeatability)
Bangalore Seetharam (2020)	4	Yes	Generic description	Early Learning, Lay Foundation, Leverage, Accelerate	N/A
Lichtenthaler (2020)	5	Yes	Detailed description	Initial Intent, Independent Initiative, Interactive Implementation, Interdependent Innovation, Integrated Intelligence	3 elements (Types of AI, AI-human intelligence interdependencies, and Meta-intelligence)
Jaaksi, Koskinen & Jalava (2018)	4	Yes	Detailed description	Level 1, Level 2, Level 3, level 4	5 dimensions (Workforce, Data management, Process, Organization status, and Organizational mentality)
Lee (2020)	5	No	Detailed description	N/A	4 elements (Data maturity, Analytics maturity, Platform maturity, and Operation maturity)
Yablonsky (2019)	5	Yes	Detailed description	Human Led; Human Led, Machine Supported; Machine Led, Human Supported; Machine Led, Human Governed; Machine Controlled	4 dimensions (Who produce insight, Who decide and how, Who acts based on decision, AA)
Burgess (2018)	6	Yes	Detailed description	Manual Processing, Traditional IT-Enabled Automation, Isolated, Basic Automation Attempts, Tactical Deployment of Individual Automation Tools, Tactical Deployment of a Range of Automation Tools, End-to-End Strategic Automation	4 capabilities (Strategic objectives, Existing challenges, Benefits, and AI Capabilities) are subdivided in 6 process areas (Customer Service, Operations, Finance, ITSM, HR, and Risk Assessment)
Ellefsen et al. (2019)	4	Yes	Very brief description	Novice, Ready, Proficient, and Advanced	N/A
Seger, Miailhe & Mueller (2019)	5	Yes	Generic description	Unaware or risk averse, Aware and resourceful, Fully developed strategic plan, AI harnessed at scale, The AIGO	N/A
Alsheibani, Cheung & Messom (2019)	5	Yes	Detailed description	Initial, Assessing, Determined, Managed, Optimized	4 dimensions (AI functions, Data structure, People, and Organization)

**Note:**

All related articles are summarized according to their maturity levels and and elements.

### The main scope of the AIMMs

This section involves answering the third research question: What are is the scope of maturity models in the AI domain? The SLR identified five AIMMs that were developed to be applicable in a generic manner. The other 10 models were created with a particular focus on a specific domain or purpose, as shown in Table 6. Five studies developed models for particular domains, while eight studies developed models for specific purposes. Two of 10 studies strived to evaluate the intelligent automation process. The first one was conducted by Tu, Lin & Lee (2019) to develop a model depending on the environment of clinical research to evaluate intelligent automation. Second, Burgess (2018) developed a model to evaluate automation maturity that focused on AI tools essentially, either at the company level or specific area.

**Table 6:** The scope of AI maturity models.

AI analysis level	Author	AI domain
Company/department	Saari, Kuusisto & Pirttikangas (2019)	General
Company	Gentsch (2018)	General
	Kreutzer & Sirrenberg (2020)	General
	Jaaksi, Koskinen & Jalava (2018)	General
	Ellefsen et al. (2019)	Logistic companies
	Seger, Miailhe & Mueller (2019)	Intergovernmental organizations
	Alsheibani, Cheung & Messom (2019)	General
Project/Company	Coates & Martin (2019)	AI bias governance
System	Abele & D’Onofrio (2020)	Intelligence
Process	Tu, Lin & Lee (2019)	Intelligent automation in Drug research
	Bangalore Seetharam (2020)	AI implementation management in Retail organization
	Lichtenthaler (2020)	AI management
	Lee (2020)	Industrial intelligent transformation in manufacturers
	Yablonsky (2019)	AI innovation management
	Burgess (2018)	Automation

**Note:**

The previous AI maturity models are grouped into five AI analysis level.

On the other hand, the AI maturity ladder was developed by Bangalore Seetharam (2020) for retail organizations. Ellefsen et al. (2019) developed a framework for evaluating AI in logistic companies. Regarding specific purposes, Coates & Martin (2019) developed a tool to evaluate AI bias governance in an entire projector company, while Yablonsky (2019) developed a framework to evaluate AI innovations as examples. Furthermore, two models were developed to evaluate the intelligence in the systems. Abele & D’Onofrio (2020) introduced a model to assess the intelligence systems based on their intelligence capabilities, while Lee (2020) developed a model to evaluate the technical aspect (AI capabilities) and provide a basic judgment about an enterprise’s intelligent transformation stage.

This review reveals four levels of analysis with a scope: a company, process, project, and system levels. One study involved analysis within the scope of the project level (Coates & Martin, 2019). Six studies (Bangalore Seetharam, 2020; Burgess , 2018; Lee, 2020; Lichtenthaler, 2020; Tu, Lin & Lee, 2019; Yablonsky, 2019) assisted organizations in assessing the single process at the process level, while only one study focused on the system level (Abele & D’Onofrio, 2020). For example, AI management (Lichtenthaler, 2020) assists an organization in achieving an integrated intelligence architecture. At the same time, a data-driven automation framework (Yablonsky, 2019) enables an organization to concentrate on the relationship between data and human-machine interactivity and the implementation of AI at different levels of data-driven automation maturity scopes. The SLR listed 10 maturity models with a company-level scope to evaluate AI maturity of the entire company. The SLR provides evidence that most of the maturity models were developed to be applicable for a particular focus on a specific domain or purpose. Additionally, most maturity models had a scope at the company level, followed by the process level.

### Design, typology, and architecture of the AIMMs

This section explores issues related to the models’ designs to answer the last research question. Not all of the studies indicate the design approach of the maturity model. Just four studies followed the bottom-up approach. No studies used a top-down approach. According to the topology, three studies followed Likert-like questionnaires as part of a quantitative approach. At the same time, one study (Burgess , 2018) used a structure like CMM to evaluate automation. Most studies used the qualitative description assessment approach (five studies followed the matrix grid). The dominant form to represent maturity is continuous representation. Only two studies used stage representation.

The final issue discussed in this section involves the benefits and purposes of use. The benefits of using maturity models could be descriptive, prescriptive, or comparative (Becker, Knackstedt & Pöppelbuß, 2009; De Bruin et al., 2005; Mettler, 2011; Poppelbub & Roglinger, 2011). The descriptive approach is used to evaluate the current situation of a domain as it is. The prescriptive approach is used to give a roadmap or vision to improve the AI in an organization. Finally, the comparative approach provides a comparison within an organization among a series of organizations. Poppelbub & Roglinger (2011) identified multiple purposes for using the same model during their analysis. This review also identified multiple purposes of use for most of the models, based on the information mentioned in the studies. For example, Gentsch (2018) explained that the model could be used to evaluate the current status with regards to AI, algorithms, and big data (descriptive), as a guideline to the next maturity level (prescriptive), and compare similar practices between organizations to evaluate maturity in different sectors (comparative). Saari, Kuusisto & Pirttikangas (2019) indicated that the final graph would compare an organization, among organizations, and with a reference group if there are sufficient respondents (comparative). In addition, it is used as a self-assessment tool (descriptive), and its result helps the organization identify the most critical areas for improvement (prescriptive). Alsheibani, Cheung & Messom (2019) indicated that AIMM could be used to assess current AI capability (descriptive), offer managers with insights to adopt AI solutions (prescriptive), and benchmark against other advanced organizations to develop their AI capabilities (comparative) gradually. Most AIMMs have a descriptive characteristic (13 articles), but there is a lower amount that use the prescriptive purpose (only six studies). In contrast, comparative approaches are largely unmet (only three papers). All the previous information is shown in Table 7.

**Table 7:** The approach design, topology, architecture, and assessment purpose of AI maturity models.

Purpose of use	Author	Design approach	Typology	Architecture
Descriptive,	Gentsch (2018)	Bottom-up	Maturity Grid	Continuous
Prescriptive, and	Saari, Kuusisto & Pirttikangas (2019)	N/A	Likert-like questionnaires	Continuous
Comparative	Alsheibani, Cheung & Messom (2019)	Bottom-up	Likert-like questionnaires	Continuous
Descriptive and Prescriptive	Kreutzer & Sirrenberg (2020)	N/A	N/A	Continuous
Prescriptive	Bangalore Seetharam (2020)	Bottom-up	N/A	N/A
	Burgess (2018)	N/A	Structured models	Stage
	Coates & Martin (2019)	N/A	Likert-like questionnaires	Stage
	Abele & D’Onofrio (2020)	N/A	N/A	Continuous
	Tu, Lin & Lee (2019)	N/A	Matrix grid	Continuous
Descriptive	Lichtenthaler (2020)	Bottom-up	N/A	Continuous
	Jaaksi, Koskinen & Jalava (2018)	N/A	Matrix grid	Continuous
	Lee (2020)	N/A	Matrix grid	Continuous
	Yablonsky (2019)	N/A	Matrix grid	Continuous
	Ellefsen et al. (2019)	N/A	N/A	Continuous
	Seger, Miailhe & Mueller (2019)	N/A	N/A	N/A

**Note:**

All previous AI maturity models are categorized into their purpose of uses based on their design approach, typology and architecture.

## Discussion

AI is an important technology that uses in different sectors. However, using it in these sectors remains at an early stage. The importance of AIMM is to evaluate the current AI situation and achieve higher quality in various companies. Therefore, this review is conducted to give knowledge about the recent publications on this topic. In the study, a final sample of 15 papers was included based on the methodologies proposed by Okoli (2015). The majority of the studies’ research objectives of AIMM focused on maturity model development, followed by validations. This finding appears to reflect a pattern observed in various domain investigations of maturity models (Wendler, 2012). It is a reflection of community dissatisfaction with existing approaches. However, the theoretical reflections of the maturity concept are mostly missing. A theoretical foundation is necessary to establish a well-founded development for a suitable and ready-to-use maturity model for practice and useful for other researchers. This leads to conclude that this topic needs more examination, especially since the number of documents is relatively small relative to other publications on maturation models in other domains. Process theory and resource-based view are an example of theories that could be used in AI research.

Most studies are empirical studies that have been conducted to develop or validate maturity models. It seems that this result usual and dominates the maturity models literature (Wendler, 2012). For designing the model, qualitative content analysis prevailed. This is normal where qualitative approaches are more used than quantitative methods when developing maturity model constructs (Lasrado, Vatrapu & Andersen, 2015). Seven (47%) out of 15 studies validated the design model, indicating that many AIMMs lack validation of their structure and applicability. However, this finding may explain why we could not find a study about the applicability of AIMMs in the sources. Some authors introduce the instrument (*i.e*., survey) to classify organizations and make some conclusions. However, the measurement tool was not tested on a sample for accuracy. Validation is the degree to which the model accurately depicts reality and considers a sequel to the design procedure (Becker, Knackstedt & Pöppelbuß, 2009). It is necessary to check the validity, reliability, and generalizability of both the model construction and the model instruments (De Bruin et al., 2005). Thus, the without validation models of AI in reality raises questions about ensuring their practical relevance and competence to identify maturity levels. On the other hand, a sufficient (empirical) validation of the model is also essential. The most validation method is mixed methods or qualitative methods. The mixed-methods approach used either mixed two qualitative methods or mixed qualitative and quantitative methods (most used approaches). This is because a mixture of quantitative and qualitative methods can provide more generalized insights (Wendler, 2012). For instance, after conducting a survey to validate the instrument by Coates & Martin (2019), case studies were followed through interviews to support the development by confirming results. Besides, it leads to attaining deep, accurate, and more reliable results. However, using two qualitative methods conducted by Gentsch (2018) is also useful because data triangulation using mixed methods will improve the model’s reliability and validity. In different papers, qualitative methods (*e.g*., case study, grounded theory) seem recommendable due to their ability to provide a deep understanding of the research’s object.

Identifying the scope is the most critical step during the design process (Mettler, 2011). Two criteria for the scoping of the model: the focus of the model, which will determine the specificity and extensibility of the model within a particular domain (Bertolini et al., 2019). The second is development stakeholders, which define the source of knowledge and the possible audience (Bertolini et al., 2019). The scope of the maturity model should be linked with the demands of stakeholders (*e.g*., practitioners, academics, non-profit organizations) (Mettler, 2011). The review reveals that only one study mentioned that the ladder was developed based on the feedback received from the stakeholders (Alsheibani, Cheung & Messom, 2019). According to the focus of the models, this study reveals that most models narrowed the scope of the models to be applicable in specific industries or to be used for particular purposes. However, most of them are at the process level. This is due to no one size of AIMM that suits all organizations in different sectors (Alsheibani, Cheung & Messom, 2019). Creating an AIMM for a specific industry provides an opportunity to collect all the dimensions in the context whereby the AIMM for generic could not cover all of them. This leads, for example, Lasrado, Vatrapu & Andersen (2015) developing the model because there is no standardized measurement norm for the field of Industrial Intelligence to assess intelligence capability in the AI processes. Although, the trends of studies were focused on the company level, and most of them developed for general purpose. This is due to AIMMs at the company level’s goal to gain a comprehensive view of the company’s strengths by aggregating multiple capability areas (*e.g*., people skills, top leadership, or organizational culture) and the company’s goals relevant to decision-making (Lichtenthaler, 2020). While at the process level scope focuses on technology issues obstructs identifying other critical elements that contribute to higher levels of AI capability. As a result, the applicability scope of the models is varied, and there is a balance between models developed at company and process levels. We believe that using AIMMs across various organizations will be beneficial, particularly for benchmarking AI capabilities. Furthermore, more studies focus on evaluating the maturity of the AI workforce (*e.g*., AI team) are needed.

Regarding the maturity model design, the SLR shows that four models use a bottom-up method, while none use a top-down approach. AI is not a new technology and uses in various organizations. This can explain the popularity of the bottom-up approach in AIMM design, which is more appropriate than a top-down approach which is more suitable if the domain is naive with little evidence to reflect its maturity (De Bruin et al., 2005). The remaining eleven papers make it difficult to define a feature that would lead to classification into either approach due to a lack of information when documenting the maturity model growth method. This finding indicates that the design approach that followed was not familiar in the maturity model.

When applying a maturity model, there are differences in the representation of the maturity level (stage or continuous) and the assessment method (matrix grid or Likert-like questionnaires). No papers identified using a hybrid of them, while only one study used a structure like CMM. This is because a hybrid or structure like CMM will increase the complexity of the model compared to the other Likert-type and maturity grids (Fraser, Moultrie & Gregory, 2002). This makes the process of determining maturity level does not present great difficulties. Six studies did not identify the topology of their models. It is obvious that continuous representation was used higher in the selected studies. The justification is presentation allows organizations to enhance their capabilities in particular process areas without allocating an area to a specific level of maturity (Fraser, Moultrie & Gregory, 2002). As a result, there is liberty in this representation to determine the sequence of improvement for the considered regions and processes.

According to the purposes of AIMMs, descriptive (13 articles) and comparative (three articles) approaches were found in 87% and 20% of the articles, respectively. These approaches have nature only identify problems or determine the *status quo* of capabilities, but neither of them indicates how to solve problems or increase the maturity level. On the other hand, only 40% reflect a prescriptive method. The low number of papers with a prescriptive approach inhibits the practical implementation, and widespread use of these models, especially AIMM attempts to give guidance or a clear roadmap for achieving a greater level of maturity. Besides, the limited references to comparative studies demonstrate the emerging nature of this field and the necessity for development.

Every maturity model consists of several maturity levels that differ from model to model (De Bruin et al., 2005). It could be somewhat arbitrary, and the higher the number of levels, the more difficult and complex it is (Fraser, Moultrie & Gregory, 2002). Every level should include a label or descriptor with descriptions to clarify the characteristics of each level as a whole (De Bruin et al., 2005; Fraser, Moultrie & Gregory, 2002). This review exposes that there is differentiation in the number of maturity levels. The most frequent number is five levels, which is suitable because it would prevent the dispersion of details and make the development process less complicated. The number of maturity model elements varies as well, from one to another. These elements represent the domain components that are needed to evaluate maturity. The number of domain components and subcomponents, and specific capability areas within the domain components, should be kept low when designing a model to reduce the perceived complexity of the model and ensure the components’ independence (De Bruin et al., 2005). This review reveals that Coates & Martin (2019) make up the most significant number of elements. This is because they developed an instrument to evaluate the AI bias governance in a project through two stages: Design and Development and Post Development.

The critical success factors identified from 13 studies are Data, Analytics, Technology and Tools, Intelligent Automation, Governance, People, and Organization, which could be considered the most critical dimensions. The elements were classified according to the explanations. Most elements in the ten studies are about evaluating intelligent automation and the intelligent levels of their systems. Only two studies concern governance, and one of them focus only on data compliance. Even the studies that developed maturity models to evaluate AI in the whole company did not pay attention to measuring it. The same conclusion to analytics is an essential dimension for developing a maturity model for the AI domain. The maturity model that does not capture all the relevant dimensions in its structure will not reflect the current AI capability situation. Furthermore, six out of 13 studies (46%) on AI maturity pertain to assess the technology aspect, even in specific domains. It confirms that organizations still require an improvement in their AI capability and in strengthening AI maturity.

The above indicates that the maturity models under review do not provide a complete image to evaluate and increase AI in companies. This finding is in line with Poppelbub & Roglinger (2011), who mentioned that new maturity models are frequently introduced in scholarly papers as a rough theoretical sketch that is not appropriate for practical use. Scholars often fall short in offering comprehensive guidance and helpful (software-based or online) toolkits to promote the practical implementation of models established in academia.

Both the research and practitioner communities may benefit from the answers to these issues. Researchers can utilize the study results as a basic and inspiration for their own future research efforts. Simultaneously, the research could serve as a useful beginning point for practitioners. They can choose which AIMMs are suited for their work or determine which maturity notion is significant for them to deliver practical results.

## Conclusions

AI is a vital technology that is used in a variety of organizations. However, its application is still at the early stages. Thus, adopting maturity models to assess the current state of AI and encourage adoption in diverse enterprises is critical. This paper examines various AIMMs used in academic studies to understand better the aims, research methods, and main characteristics of maturity models. Most of the papers’ objectives are on the maturity model development (RQ1). Seven (47%) out of 15 studies have rigorously validated, focusing on mixed methods and qualitative methods. (RQ2). Out of 15 studies, 10 (67%) are designed for specific domains and purposes, while 12 studies (80%) their scope’s levels are company and process (RQ3). The trend of models’ design is a bottom-up design approach, maturity grid, and continuous representation with five levels and focus on descriptive purposes in the papers. The critical success factors identified from 13 studies are Data, Analytics, Technology and Tools, Intelligent Automation, Governance, People, and Organization, which could be considered the most critical dimensions for an organization. These results provide an answer to the last research question (RQ4).

No studies have paid attention to measure all the critical dimensions, even models designed on the company level. The model that does not capture all the critical dimensions will not reflect the current AI capability situation. Furthermore, a minimal number of works simultaneously study maturity models and AI, illustrating the need further to explore this research field with a theoretical background and collect all the critical dimensions to ensure that the organization or process situation is faithfully assessed. This research has some limitations. More research is required, either in other libraries or by using different keywords like (assessment maturity and AI or maturity matrix and AI) to identify more relevant papers.

## Additional Information and Declarations

### Data Availability

The following information was supplied regarding data availability: This is a systematic literature review; there are no raw data or code.

## References

[ref-60] Abd Hamid A, Mansor Z (2016). Clients readiness assessment success factors for outsourcing software projects. International Journal on Advanced Science, Engineering and Information Technology.

[ref-47] Abdullah MF, Ahmad K (2015). Business intelligence model for unstructured data management.

[ref-2] Abele D, D’Onofrio S (2020). Artificial intelligence—the big picture.

[ref-3] Alsheibani S, Cheung Y, Messom C (2019). Towards an artificial intelligence maturity model: from science fiction to business facts.

[ref-4] Bahri S, Fauzi A (2018). Developing a maturity model for government community broadband projects. International Journal of Electronic Governance.

[ref-49] Bakar H, Razali R, Jambari DI (2020). A guidance to legacy systems modernization. International Journal on Advanced Science, Engineering and Information Technology.

[ref-5] Bangalore Seetharam S (2020). *Developing a digital AI roadmap for retail, Master dissertation Thesis*.

[ref-6] Becker J, Knackstedt R, Pöppelbuß J (2009). Developing maturity models for IT management. Business & Information Systems Engineering.

[ref-57] Bertolini M, Esposito G, Neroni M, Romagnoli G (2019). Maturity models in industrial internet: a review. Procedia Manufacturing.

[ref-1] Burgess A (2018). Starting an AI journey.

[ref-7] Coates DL, Martin A (2019). An instrument to evaluate the maturity of bias governance capability in artificial intelligence projects. IBM Journal of Research and Development.

[ref-8] Dahlan HA (2018). Future interaction between man and robots from Islamic perspective. International Journal of Islamic Thought.

[ref-9] De Bruin T, Rosemann M, Freeze R, Kaulkarni U (2005). Understanding the main phases of developing a maturity assessment model.

[ref-10] Dzazali S, Zolait AH (2012). Assessment of information security maturity: an exploration study of Malaysian public service organizations. Journal of Systems and Information Technology.

[ref-11] Ellefsen APT, Oleśków-Szłapka J, Pawłowski G, Toboła A (2019). Striving for excellence in AI implementation: AI maturity model framework and preliminary research results. LogForum.

[ref-12] Felch V, Asdecker B, Sucky E (2019). Maturity models in the age of Industry 4.0—Do the available models correspond to the needs of business practice?. HICSS.

[ref-13] Fraser P, Moultrie J, Gregory M (2002). The use of maturity models/grids as a tool in assessing product development capability.

[ref-14] Frick N, Küttner TF, Schubert P (2013). Assessment methodology for a maturity model for interorganizational systems—the search for an assessment procedure.

[ref-15] Gemmink M (2019). The adoption of reinforcement learning in the logistics industry: a case study at a large international retailer.

[ref-16] Gentsch P (2018). AI business: framework and maturity model. AI in Marketing, Sales and Service.

[ref-17] Greenhalgh T, Peacock R (2005). Effectiveness and efficiency of search methods in systematic reviews of complex evidence: audit of primary sources. BMJ.

[ref-18] Haenlein M, Kaplan A (2019). A brief history of artificial intelligence: on the past, present, and future of artificial intelligence. California Management Review.

[ref-50] Hassan E, Yusof ZM, Ahmad K (2019). Factors affecting information quality in the malaysian public sector. International Journal of Advanced Science Engineering Information Technology.

[ref-19] Herbsleb JD, Goldenson DR (1996). A systematic survey of CMM experience and results.

[ref-20] Jaaksi J, Koskinen J, Jalava M (2018). How To Define An Organization’s Maturity For Adopting Artificial Intelligence Solutions. Master dissertation Thesis.

[ref-58] Keele S (2007). Guidelines for performing systematic literature reviews in software engineering.

[ref-21] Khaki M, Yusoff I, Islami N, Hussin NH (2016). Artificial neural network technique for modeling of groundwater level in Langat Basin, Malaysia. Sains Malaysiana.

[ref-22] Kiat PE, Malek MA, Shamsuddin SM (2019). Artificial intelligence projection model for methane emission from livestock in Sarawak. Sains Malaysiana.

[ref-23] Kılınç İ, Ünal A (2019). AI is the new black: effects of artificial intelligence on business world. Journal of Contemporary Administrative Science.

[ref-24] Kohlegger M, Maier R, Thalmann S (2009). Understanding maturity models. Results of a structured content analysis.

[ref-25] Kreutzer RT, Sirrenberg M (2020). AI challenge—how artificial intelligence can be anchored in a company.

[ref-26] Kumar R (2017). Machine learning and cognition in enterprises: business intelligence transformed.

[ref-48] Kurniawan R, Nazri MZA, Abdullah SNHS, Murhayati S (2018). Using bayesian network for determining the recipient of zakat in BAZNAS pekanbaru.

[ref-27] Lamberti MJ, Wilkinson M, Donzanti BA, Wohlhieter GE, Parikh S, Wilkins RG, Getz K (2019). A study on the application and use of artificial intelligence to support drug development. Clinical Therapeutics.

[ref-28] Lasrado LA, Vatrapu R, Andersen KN (2015). Maturity models development in is research: a literature review.

[ref-29] Lee J (2020). How to establish industrial AI technology and capability. Industrial AI.

[ref-30] Lichtenthaler U (2020). Five maturity levels of managing AI: from isolated ignorance to integrated intelligence. Journal of Innovation Management.

[ref-31] Martín-Martín A, Thelwall M, Orduna-Malea E, Delgado López-Cózar E (2021). Google scholar, microsoft academic, scopus, dimensions, web of science, and OpenCitations’ COCI: a multidisciplinary comparison of coverage via citations. Scientometrics.

[ref-32] Mettler T (2011). Maturity assessment models: a design science research approach. International Journal of Society Systems Science.

[ref-33] Mikalef P, Fjørtoft SO, Torvatn HY (2019). Artificial Intelligence in the public sector: a study of challenges and opportunities for Norwegian municipalities.

[ref-34] Mohammad M, Mann R, Grigg N, Wagner JP (2009). Selection of quality improvement initiatives: an initial conceptual model. Journal of Quality Measurement and Analysis.

[ref-53] Moher D, Shamseer L, Clarke M, Ghersi D, Liberati A, Petticrew M, Shekelle P, Stewart LA (2015). Preferred reporting items for systematic review and meta-analysis protocols (PRISMA-P) 2015 statement. Systematic Reviews.

[ref-35] Nortje M, Grobbelaar S (2020). A framework for the implementation of artificial intelligence in business enterprises: a readiness model.

[ref-52] Ochoa-Urrego R-L, Peña-Reyes J-I, Schallmo DRA, Tidd J (2021). Digital maturity models: a systematic literature review. Digitalization. Management for Professionals.

[ref-36] Okoli C (2015). A guide to conducting a standalone systematic literature review. Communications of the Association for Information Systems.

[ref-37] Paulk MC (2009). A history of the capability maturity model for software. ASQ Software Quality Professional.

[ref-38] Poppelbub J, Roglinger M (2011). What makes a useful maturity model? A framework of general design principles for maturity models and its demonstration in BPM.

[ref-40] Saari L, Kuusisto O, Pirttikangas S (2019). VTT Technical Research Centre of Finland. VTT White Paper.

[ref-54] Santos-Neto JBSd, Costa APCS (2019). Enterprise maturity models: a systematic literature review. Enterprise Information Systems.

[ref-41] Seger J, Miailhe N, Mueller S (2019). *The AIGO: A Framework for Planning, Developing, and Deploying Artificial Intelligence in Intergovernmental Organizations. Research Report. The Future Society. Harvard Kennedy School of Government*.

[ref-55] Tarhan A, Turetken O, Reijers HA (2016). Business process maturity models: a systematic literature review. Information and Software Technology (IST).

[ref-59] Tocto-Cano E, Paz Collado S, López-Gonzales JL, Turpo-Chaparro JE (2020). A systematic review of the application of maturity models in universities. Information.

[ref-51] Tranfield D, Denyer D, Smart P (2003). Towards a methodology for developing evidence‐informed management knowledge by means of systematic review. British Journal of Management.

[ref-42] Tu H, Lin Z, Lee K (2019). Automation with intelligence in drug research. Clinical Therapeutics.

[ref-43] Wacker JG (1998). A definition of theory: research guidelines for different theory-building research methods in operations management. Journal of Operations Management.

[ref-56] Webster J, Watson RT (2002). Analyzing the past to prepare for the future: writing a literature review. MIS quarterly.

[ref-44] Wendler R (2012). The maturity of maturity model research: a systematic mapping study. Information and Software Technology.

[ref-45] Yablonsky S (2019). Multidimensional data-driven artificial intelligence innovation. Technology Innovation Management Review.

[ref-46] Zhang H, Song M, He H (2020). Achieving the success of sustainability development projects through big data analytics and artificial intelligence capability. Sustainability.

